# Polydatin up-regulates clara cell secretory protein to suppress phospholipase A2 of lung induced by LPS in vivo and in vitro

**DOI:** 10.1186/1471-2121-12-31

**Published:** 2011-07-25

**Authors:** Shu Shiyu, Ling Zhiyu, Ye Mao, Bo Lin, Wang Lijia, Zhang Tianbao, Chen Jie, Li Tingyu

**Affiliations:** 1 Department of Anesthesiology, Children's Hospital of Chongqing Medical University, Zhongshan Er Road NO136, Yuzhong District, Chongqing 40 0014, China; 2 Department of Cardiology, Second Affiliated Hospital of Chongqing Medical University, Linjiang Road NO74, Yuzhong District, Chongqing 40 0010, China; 3 Immunological laboratory, Ministry of Education Key Laboratory of Child Development and Disorders; Zhongshan Er Road NO136, Yuzhong District, Chongqing 40 0014, China; 4 Department of Health and Toxicology, Second Military Medical University of PLA, Xiangyin Road NO800, Shanghai 20 0433, China; 5 Children Nutrition Research Center, Key Laboratory of Pediatrics in Chongqing, CSTC2009CA 5002, Zhongshan Er Road NO136, Yuzhong District, Chongqing 40 0014, China; 6 Chongqing International Science and Technology Cooperation Center for Child Development and Disorders, Zhongshan Er Road NO136, Yuzhong District, Chongqing 40 0014, China

## Abstract

**Background:**

Lung injury induced by lipopolysaccharide (LPS) remains one of the leading causes of morbidity and mortality in children. The damage to membrane phospholipids leads to the collapse of the bronchial alveolar epithelial barrier during acute lung injury (ALI)/acute respiratory distress syndrome (ARDS). Phospholipase A_2 _(PLA_2_), a key enzyme in the hydrolysis of membrane phospholipids, plays an important traumatic role in pulmonary inflammation, and Clara cell secretory protein (CCSP) is an endogenous inhibitor of PLA_2_. Our previous study showed that polydatin (PD), a monocrystalline extracted from a traditional Chinese medicinal herb (Polygonum cuspidatum Sieb, et Zucc), reduced PLA_2 _activity and sPLA_2_-IIA mRNA expression and mitigated LPS-induced lung injury. However, the potential mechanism for these effects has not been well defined. We have continued to investigate the effect of PD on LPS-induced expression of CCSP mRNA and protein in vivo and in vitro.

**Results:**

Our results suggested that the CCSP mRNA level was consistent with its protein expression. CCSP expression was decreased in lung after LPS challenge. In contrast, PD markedly increased CCSP expression in a concentration-dependent manner. In particular, CCSP expression in PD-pretreated rat lung was higher than in rats receiving only PD treatment.

**Conclusion:**

These results indicated that up-regulation of CCSP expression causing inhibition of PLA_2 _activation may be one of the crucial protective mechanisms of PD in LPS-induced lung injury.

## Background

Acute lung injury (ALI), or its severe form, acute respiratory distress syndrome (ARDS), induced by sepsis is still a major cause of morbidity and mortality in children [1]. ALI is characterized by an extensive neutrophil influx into the lung, the expression of proinflammatory mediators and damage to the lung epithelium and endothelium. Current clinical and experimental research on the treatment of lung injury is aimed at inhibiting different stages of this process with drugs or therapy, along with enhancing the body's own resistance, to delay or mitigate lung injury. However, the outcome of sepsis and septic shock cases has not been improved significantly. Mortality in ALI is still as high as 18%-27%, and the mortality rate of (ARDS) is even higher to 29%-50% [2]. Therefore, improved treatments and prevention strategies are needed to minimize the mortality associated with ALI.

It is generally acknowledged that damage to membrane phospholipids leads to the collapse of the bronchial alveolar epithelial barrier during ALI/ARDS. Phospholipase A_2 _(PLA_2_), a key enzyme that hydrolyzes membrane phospholipids, plays a critical traumatic role in pulmonary inflammation through its influence on membrane signal transduction, biomembrane stability, activation of lipid mediators and leukocyte-endothelial cell adhesion cascade formation. PLA_2 _hydrolyzes the fatty acid from the sn-2 position of phospholipids to release arachidonic acid, prostaglandins, platelet-activating factor and other inflammatory mediators [3]. In mammals, PLA_2 _forms a large family of enzymes that can be schematically divided into two major classes: high-molecular-weight intracellular PLA_2 _(cPLA_2_) and low-molecular-weight secretory PLA_2_(sPLA_2_), including sPLA_2_-IIA. The former (cPLA_2_) is now generally considered to be a central enzyme mediating generation of eicosanoids and hence many inflammatory processes. The latter (sPLA_2_) is found at high levels in the circulation and locally in the tissues and has been suggested to play a role in a number of inflammatory diseases by regulating the synthesis of prostaglandins, leukotrienes and platelet activating factor[4]. Especially, recent study showed that sPLA_2_-IIA catalyzes the hydrolysis of surfactant phospholipids and suggested that this process can contribute to the loss of surface tension-lowering properties of surfactant [5]. Clinically, sPLA2-IIA offers new possibilities as an early marker for severe inflammation and predicting systemic complications in severely ill patients. Thus, PLA_2 _is regarded as the core and rate-limiting enzyme of inflammation and plays an important role in the pathogenesis of LPS-induced acute lung injury. Specific inhibitors can be used to elucidate the roles of PLA_2 _in cellular processes or to develop new potential therapeutics [6]. Sato R reported [7] that LY37 4388, an exogenous inhibitor of sPLA_2_, may exert a protective effect on LPS-induced acute lung injury in male C57BL/6J mice.

Clara cell secretory protein (CCSP; also known as CC16, CC10 and uteroglobin) is a 16-kDa homodimeric protein that is secreted by non-ciliated bronchiolar (Clara) cells into the mucus lining the bronchial epithelium of the mammalian lung. It is one of the most abundant proteins in the airway mucus of mammals [8]. As a biomarker of Clara cells and lung health, CCSP has been proposed as a useful diagnostic marker of toxicant exposure or airway epithelial damage. Some studies have reported that administration of exogenous CCSP such as recombinant human CCSP to the lungs mitigates inflammation [9-11]. Although evidence points to antioxidant [12], anti-inflammation, immunomodulation, anti-cytokine, pollutant clearance, anti-fibrosis, anti-tumor invasion and anti-protease activity [13], its endogenous mechanism is not known. Studies on CCSP-deficient mice have indicated that CCSP acts as an endogenous inhibitor of PLA_2 _[14,15] by binding calcium, a cofactor for secretory PLA_2 _activation, or phosphatidylcholine, a substrate of PLA_2 _[16].

**Figure 1:**
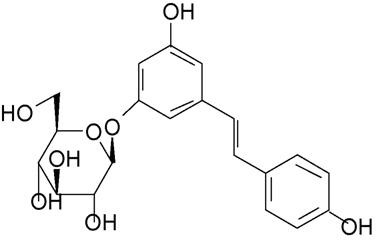
**The chemical structure of trans-polydatin (3,4',5-trihydroxystilbene-3-β-single-D-glucoside)**.

Polydatin (PD), also known as polygonin, is a type of polyphenolic phytoalexin with strong antioxidative activity that is isolated from a variety of plant species and also can be readily synthesized [17,18]. One of the richest sources of this monocrystalline compound is the roots of Polygonum cuspidatum Sieb, a weed widely used in traditional Chinese and Japanese medicine. Its chemical name is 3, 4', 5-trihydroxystilbene-3-β-single-D-glucoside [19] (Figure 1). PD exhibits multiple biological activities and pharmacological effects, such as protection of the heart [19,20], brain [21] and liver [22], improving microcirculation [23], inhibiting platelet aggregation [24], combating shock [25], anti-asthmatic, microbial resistance, and anti-cancer activities. In 2006, PD was evaluated by the State Food and Drug Administration of China and became one of the new drugs approved for further testing in phase II clinical trials.

Our previous experimental results indicate that polydatin has prophylactic and therapeutic effects (the former is more distinct than the latter) on acutely injured lungs in rats with endotoxic shock by inhibiting phospholipase A_2 _activity and the gene expression of secretory phospholipase A_2 _type II A (sPLA_2_-IIA) [26]. Phospholipase A_2 _activity and sPLA_2_-IIA expression are increased by endotoxin injection, but treatment with polydatin inhibits these increases. In our preliminary experiments, obvious morphological evidence was found in pathological lung sections, and the protective effect of PD was most obvious in lung from rats pretreated with PD. These early results suggest that PD may act as a phospholipase A_2 _inhibitor to inhibit the activation and activity of PLA_2_. However, the potential mechanism is not yet fully understood. Therefore, the purpose of this study was to determine whether polydatin can up-regulate the expression of CCSP and inhibit PLA_2 _to suppress LPS-induced lung injury.

## Methods

### In vivo experiments

#### Animals and grouping

Ninety-eight healthy, male, 7-week-old Sprague-Dawley rats (clean grade) weighing 200-250 g were purchased from the Animal Experiment Center of Chongqing Medical University (Chongqing, China). They were housed in a regulated environment (24 ± 2°C), with a 12 hours dark and 12 hours light cycle. All animal treatments were strictly in accordance with international ethical guidelines and the National Institutes of Health Guide for the Care and Use of Laboratory Animals, and the experiments were carried out with the approval of the Committee of Experimental Animal Administration of the University.

The rats were randomly divided into five groups (Table 1): the sham operation group (I), endotoxic shock group (II), PD treatment group (III), PD pretreatment group (IV) and PD control group (V) (n = 10 each). The rats in group I were injected with normal saline and served as the control; the rats in group II were injected with LPS (10 mg per kg body weight). For group III and group IV, LPS (10 mg per kg body weight) was injected, and PD solution was injected 1 hour later or 0.5 hours before, respectively. The rats in group V received PD solution only.

**Table 1:** Animal grouping and drug injection

Group	0 h	0.5 h	1 h	2 h	6 h
I	begin	NS	NS	NS	sacrifice
II	begin	NS	LPS	NS	sacrifice
III	begin	NS	LPS	PD	sacrifice
IV	begin	PD	LPS	NS	sacrifice
V	begin	PD	NS	NS	sacrifice

PD was administered as a 0.5% solution (0.2 ml per kg body weight) with a final concentration of 1 mg PD per kg body weight.

The PD treatment and pretreatment groups were each further divided into four subgroups (n = 6 each) that received 1 mg·kg^-1^, 5 mg·kg^-1^, 10 mg·kg^-1 ^or 30 mg·kg^-1 ^PD, respectively.

#### Animal model

Rats were fasted overnight but given free access to water, then anesthetized with 20% urethane (5-6 ml·kg^-1 ^body weight) intraperitoneally. The left common carotid artery was separated and catheterized. The canal was connected to an MS302 biology signal recording and analyzing system (provided by Guangdong Pharmacological Institute in Guangzhou, China) to measure the mean arterial pressure (MAP). The right cervical vein was then separated and catheterized. LPS (Sigma, 10 mg per kg body weight) was injected from the right cervical vein. After injection, MAP fell from normal to shock levels (The blood pressure of rat and human being are almost same). After six hours' observation, they were sacrificed with cervical dislocation and their lung tissues and blood were collected for experiments.

#### Determining the expressions of CCSP/sPLA2/cPLA2 mRNA in rat lung with real-time PCR

Total RNA was isolated from lung tissue using Trizol reagent (Invitrogen). RNA was further purified with the RNeasy Mini Kit, treated with DNase I (30 U·μg^-1^total RNA) (Qiagen), and then reverse transcribed using the SuperScript III First-Strand Synthesis System (Invitrogen) according to the manufacturer's recommended protocols. Quantitative gene expression analysis was performed using the ABI Prism 7000 Sequence Detection System (Applied Biosystems), with cDNA equivalent to 10 ng of total RNA and SYBR Green PCR Master Mix (Applied Biosystems) following the manufacturer's protocol. The following primers (Table 2) were used to amplify CCSP, sPLA_2_, cPLA_2 _and β-actin.

**Table 2:** PCR primer pairs used amplify CCSP, sPLA2, cPLA2 and β-actin cDNA fragments of rat lung

Target	Oligonucleotide sequence	Tm (°C)	bp
β-actin	F: 5'-AGAGGGAAATCGTGCGTGAC-3'	60	195
	R: 5'-CCATACCCAGGAAGGAAGGCT-3'		
CCSP	F: 5'-CGGACATCTGCCCAGGATTTCT-3'	60	209
	R: 5'-ACACAGAGGACTTGTTAGGAT-3'		
sPLA2	F: 5'-CCCCAAGGATGCCACAGATT-3'	60	201
	R: 5'-TTCCGGGCAAAACATTCAGC-3'		
cPLA2	F: 5'-GAAGTTTGCTCATGCCCAGACCT-3'	60	234
	R: 5'-TTCATAGAGCGCCTTCATCACACC-3'		

F, forward primer; R, reverse primer; Tm, melting temperature.

#### Determining the expression of CCSP mRNA in rat lung with RT-PCR

Total RNA was extracted from lung tissue using Trizol reagent, and the first strand of cDNA was synthesized using reverse transcriptase. The genes of interest were then amplified by PCR. Primers corresponding to the genes of interest were the same as those mentioned previously.

#### Determining the content of CCSP protein in rat lung with western blotting

Human CCSP (Biovendor) was used as the half-quantitation standard. Protein was isolated from tissues that were homogenized in a buffer (containing protein lysates). The quantity of protein from the lung homogenate depended on the size of lung issue we used. The concentration of total protein was 10 μg·μl^-1^. The total protein of each sample for measuring was 10 μg. Samples were then combined with loading buffer containing 10% glycerol, 10% 2-mercapthanol, 2% SDS, and bromophenol blue in 0.07 M Tris-HCL (pH 6.8, boiled for 5 minutes), resolved by SDS-PAGE and then transferred to a membrane for western blotting. The membrane was incubated with a blocking solution (5% skim milk) at 24°C for 2 hours. A primary antibody was then added to the solution and incubated at 4°C for 12 hours. A peroxidase-conjugated secondary antibody solution was used to incubate the membrane at 24°C for 1 hour. The membranes were then incubated with a chemiluminescent substrate (Thermo 34077) and exposed to film (Millipore IPVH00 10).

#### Determining the concentration of CCSP protein in rat serum with ELISA

Four milliliters of blood was collected from the aorta, mixed with 1% heparin to anticoagulate for 10-20 minutes and then centrifuged for 20 minutes (2000-3000 × g). Supernatant was carefully collected for determination. The concentration of CCSP protein was measured by ELISA according to the manufacturer's instructions (Rat CCSP ELISA kit, Westang, F15255; Rabbit polyclonal to Uteroglobin, ab4 0873, Abcam).

#### Measuring CCSP positive cells with immunohistochemistry

Biopsy samples were fixed by immersion in Bouin's fluid, paraffin-embedded and cut into 6-μm-thick sections. CCSP-immunoreactive cells were detected with the uteroglobin antibody (Abcam, ab40873) and the immunoperoxidase technique. The number of CCSP-positive and -negative bronchiolar cells was determined for each bronchiolar profile examined in the lung biopsies by counting all surface epithelial cells on the bronchial biopsy specimens. For each sample, the proportion of CCSP-positive cells was expressed as a percentage of the whole bronchiolar epithelium cell population examined.

### In vitro experiments

#### Cells culture

All cell lines used in this study were purchased from the American Type Culture Collection (Manassas, VA). BEAS-2B cells are human bronchial epithelia cells transformed by the SV40 T-antigen. BEAS-2B cells were cultured in LHC-8 medium (Gibco, America) and incubated at 37°C in an atmosphere of 5% CO_2 _and 95% air. The medium was replaced every second day, and cells were passaged when > 85% confluent by washing with Ca^2+^- and Mg^2+^-free PBS and dislodging with 0.05% trypsin. MTT assay was used to determine the suitable concentrations of PD and LPS.

#### Cells grouping

Cells were divided into five groups: the normal control group, containing cultured BEAS-2B cells under normal conditions for 28 hours; the LPS group, containing cultured BEAS-2B cells in LHC-8 medium treated with LPS (100 ng·ml^-1^) for 28 hours to mimic a LPS-induced inflammatory response model; the PD treatment group, containing cultured BEAS-2B cells challenged with LPS (100 ng·ml^-1^) in LHC-8 medium for 24 h, followed by PD treatment (0.5 m mol·L^-1 ^in LHC-8 medium) for 4 h; the PD pretreatment group, containing cultured BEAS-2B cells that were pretreated with PD (0.5 m mol·L^-1^) in LHC-8 medium for 4 hours, followed by an LPS challenge (100 ng·ml^-1^) in LHC-8 medium under normal conditions for 24 hours; and the PD control group, containing cultured BEAS-2B cells treated with PD (0.5 m mol·L^-1^) in LHC-8 medium for 28 hours.

#### Determining the expression of CCSP mRNA in BEAS-2B cells

Total RNA was isolated from cells using Trizol reagent (Invitrogen) according to the manufacturer's instructions. RNA was further purified with an RNeasy Mini Kit and treated with DNase I (30 U·μg^-1 ^total RNA) then reverse-transcribed using the Super Script III First-Strand Synthesis System according to the protocols supplied by the manufacturer. Quantitative gene expression analysis was performed using the ABI Prism 7000 Sequence Detection System, with cDNA equivalent to 10 ng of total RNA and SYBR Green PCR Master Mix following the manufacturer's protocol. The following primers (Table 3) were used to amplify CCSP and β-actin.

**Table 3:** PCR primer pairs used amplify CCSP and β-actin cDNA fragments of BEAS-2B cells

Target	Oligonucleotide sequence	Tm (°C)	bp
CCSP	F: 5'- GAAACTCGCTGTCACCCTCACC-3'	60	211
	R: 5'-TTAATGATGCTTTCTCTGG-3'		
β-actin	F: 5'-CCTGTACGCCAACACAGTGC-3';	60	234
	R: 5'-ATACTCCTGCTTGCTGATCC-3'		

F, forward primer; R, reverse primer; Tm, melting temperature.

#### Determining the expression of CCSP protein in BEAS-2B

Cells were washed with phosphate-buffered saline and lysed in lysis buffer. Lysates were subjected to 4% to 15% SDS-PAGE and western blotting with anti-c-kit (1:100) antibody. Immunopositive bands were visualized by enhanced chemiluminescence (ECL, Amersham). Blots were stripped and reprobed with GAPDH antibody (1:2000) as a control to ensure equal loading.

### Statistical analysis

All data were expressed as the mean ± SEM. Data were analyzed with SSPS for Windows 13.0 using one-way analysis of variance (ANOVA) and the Bonferroni correction for multiple comparisons, when appropriate. In all cases, P values less than 0.05 was considered statistically significant.

## Results

### Polydatin up-regulates CCSP mRNA expression in rat lung

**Figure 2:**
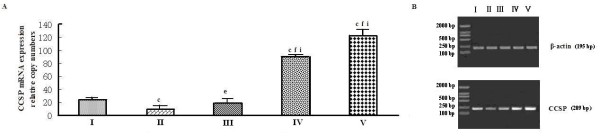
**Effects of polydatin on the expression of CCSP mRNA in rat lung**. A. Relative expression of CCSP, quantified by real-time PCR. B. Electrophoresis showing the RT-PCR results. I: sham operation group, injected with normal saline; II: endotoxic shock group, injected with LPS (10 mg·kg^-1 ^body weight); III: PD treatment group, injected with LPS (10 mg·kg^-1 ^body weight), then a 0.5% PD solution (0.2 ml·kg^-1 ^body weight) 1 hour later; IV: PD pretreatment group, injected with a 0.5% PD solution (0.2 ml·kg^-1 ^body weight), then LPS (10 mg·kg^-1 ^body weight) 0.5 hours later; V: PD control group. ^c^P < 0.01 vs. group I; ^e^P < 0.05 and ^f^P < 0.01 vs. group II; ^i^P < 0.05 vs. group III.

**Figure 3:**
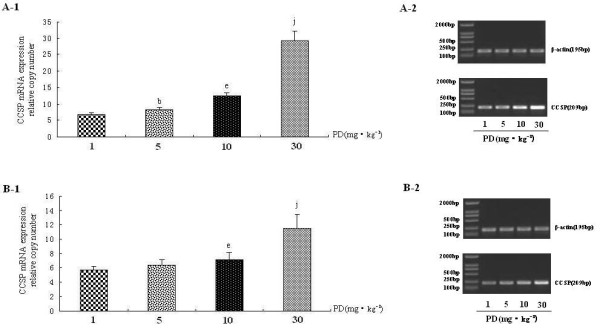
**The dose-effect relationship between polydatin and CCSP mRNA expression in rat lung**. A. CCSP mRNA expression in PD treatment groups with different doses by real-time PCR (A-1) and RT-PCR(A-2). B. CCSP mRNA expression in PD pretreatment groups with different doses by real-time PCR (B-1) and RT-PCR(B-2). ^b^P < 0.05 vs. 1 mg·kg^-1 ^PD group, ^e^P < 0.05 vs. 5 mg·kg^-1^PD group, and ^j^P < 0.01 vs. 10 mg·kg^-1 ^PD group.

A significant decrease CCSP mRNA expression was observed only in the endotoxic shock group five hours after LPS injection (Figure 2A/2B-group II). PD can increase CCSP mRNA expression not only in normal rat lung (Figure 2A/2B, group V vs. group I) but also in rat lung following endotoxic shock(Figure 2A/2B-group III/IV). Moreover, CCSP mRNA level in the PD pretreatment group was significantly higher (P < 0.05) than in the PD treatment group (Figure 2A/2B-group III/IV, group IV vs. group III). PD increased CCSP mRNA expression in a dose-dependent manner (Figure 3A-1, A-2, B-1 and 3B-2).

### Polydatin down-regulates sPLA2 and cPLA2 mRNA expression in rat lung

**Figure 4:**
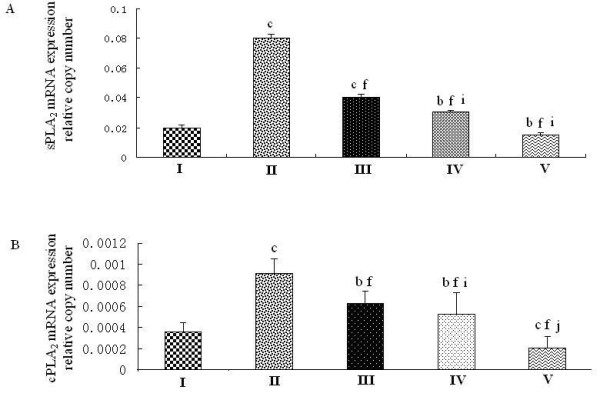
**Effects of polydatin on the expression of sPLA_2_/cPLA_2 _mRNA in rat lung**. A. Relative expression of sPLA_2_, quantified by real-time PCR. B. Relative expression of cPLA_2_, quantified by real-time PCR. I: sham operation group, injected with normal saline; II: endotoxic shock group, injected with LPS (10 mg·kg^-1 ^body weight); III: PD treatment group, injected with LPS (10 mg·kg^-1 ^body weight), then a 0.5% PD solution (0.2 ml·kg^-1 ^body weight) 1 hour later; IV: PD pretreatment group, injected with a 0.5% PD solution (0.2 ml·kg^-1 ^body weight), then LPS (10 mg·kg^-1 ^body weight) 0.5 hours later; V: PD control group. ^b^P < 0.05 and ^c^P < 0.01 vs. group I; ^f^P < 0.01 vs. group II; ^i^P < 0.05 and ^j^P < 0.01 vs. group III.

**Figure 5:**
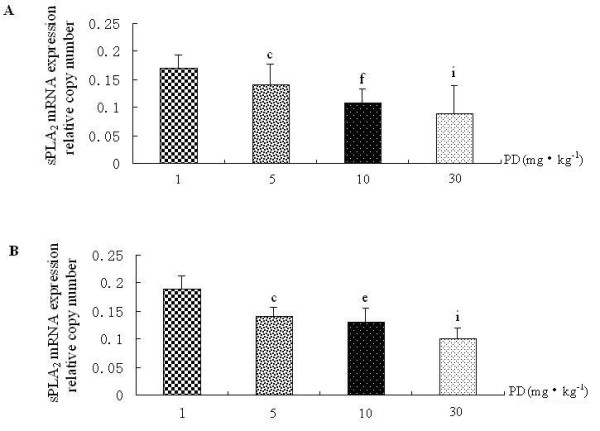
**The dose-effect relationship between polydatin and sPLA_2 _mRNA expression in rat lung**. A. sPLA2 mRNA expression in PD treatment groups with different doses by real-time PCR and RT-PCR. B. sPLA2 mRNA expression in PD pretreatment groups with different doses by real-time PCR and RT-PCR. ^c^P < 0.01 vs. 1 mg·kg^-1 ^PD group, ^e^P < 0.05 and ^f^P < 0.01 vs. 5 mg·kg^-1^PD group, and ^i^P < 0.05 vs. 10 mg·kg^-1 ^PD group.

**Figure 6:**
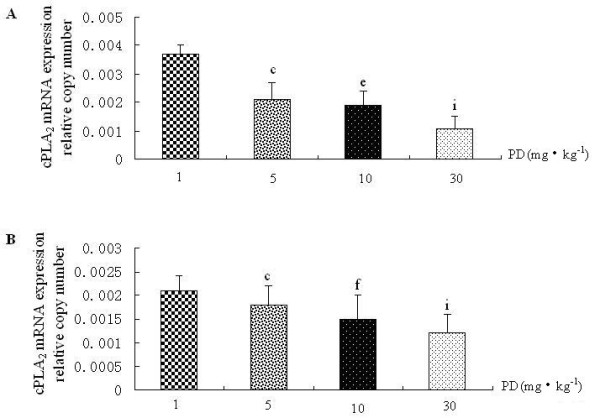
**The dose-effect relationship between polydatin and cPLA_2 _mRNA expression in rat lung**. A. cPLA_2 _mRNA expression in PD treatment groups with different doses by real-time PCR. B. cPLA_2 _mRNA expression in PD pretreatment groups with different doses by real-time PCR. ^c^P < 0.01 vs. 1 mg·kg^-1 ^PD group, ^e^P < 0.05 and ^f^P < 0.01 vs. 5 mg·kg^-1^PD group, and ^i^P < 0.05 vs. 10 mg·kg^-1 ^PD group.

Because CCSP was the potential inhibitor of PLA_2_, we investigated the effects of PD on PLA_2 _along with CCSP. PD could down-regulate the levels of sPLA_2 _and cPLA_2 _mRNA expressions in normal rat lung (Figure 4A/4B, group V vs. group I). Not only that, PD could decrease the levels of sPLA_2 _and cPLA_2 _mRNA expressions of rat lung induced by LPS (Figure 4A/4B-group III/IV), in a dose-dependent manner (Figure 5A, B and Figure 6A, B). The expression of sPLA_2 _and cPLA_2 _in PD pretreatment group was lower than in PD treatment group (Figure 4A and 4B, group IV vs. group III, P < 0.05).

### Polydatin up-regulates CCSP protein expression in rat lung

**Figure 7:**
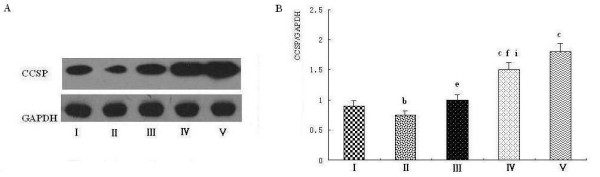
**Effects of polydatin on the expression of CCSP protein in rat lung**. CCSP protein expression was examined by western blot. I: sham operation group; II: endotoxic shock group; III: PD treatment group; IV: PD pretreatment group; V: PD control group. A. The electrophoretogram of CCSP protein expression. B. Relative ratio of CCSP protein expression. ^b^P < 0.05 and ^c^P < 0.01 vs. group I; ^e^P < 0.05 and ^f^P < 0.01 vs. group II; ^i^P < 0.05 vs. group III.

**Figure 8:**
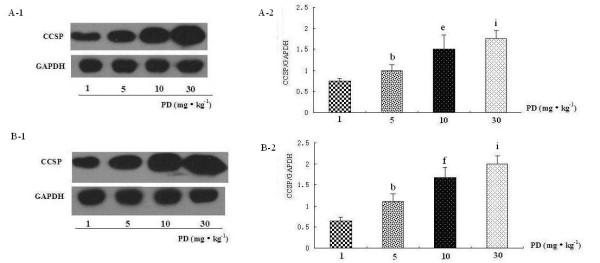
**Dose-effect relationship between polydatin and CCSP protein expression in rat lung**. A.CCSP protein expression in PD treatment groups with different doses. B.CCSP protein expression in PD pretreatment groups with different doses. ^b^P < 0.05 vs. 1 mg·kg^-1 ^PD group, ^e^P < 0.05 and ^f^P < 0.01 vs. 5 mg·kg^-1^PD group, and ^i^P < 0.05 vs. 10 mg·kg^-1 ^PD group.

To investigate whether CCSP protein expression changed with its mRNA expression synchronously, we detected CCSP protein in lung by western blot. The results indicated that LPS not only inhibited the protein expression of CCSP but also that PD treatment and PD pretreatment increased the level of CCSP markedly (Figure 7A and 7B). In particular, CCSP protein in the pretreatment and PD control groups was significantly higher than in the sham operation group (Figure 7A/7B, group IV and V vs. group I). CCSP protein expression in the PD pretreatment group was higher than in PD treatment group (Figure 7A/7B, group IV vs. group III). Thus, PD increased CCSP protein expression in a dose-dependent manner (Figure 8A-1, A-2, B-1 and 8B-2).

### Polydatin down-regulates serum CCSP level in rat

**Figure 9:**
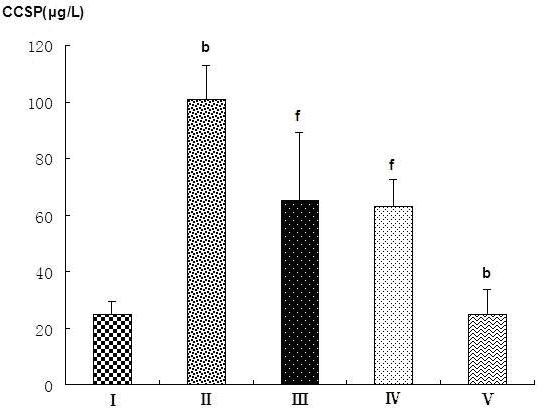
**Effects of polydatin on serum CCSP level in different groups of rats**. I: Sham operation group; II: endotoxic shock group; III: PD treatment group; IV: PD pretreatment group; V: PD control group. ^b^P < 0.05 vs. group I; ^f^P < 0.01 vs. group II.

**Figure 10:**
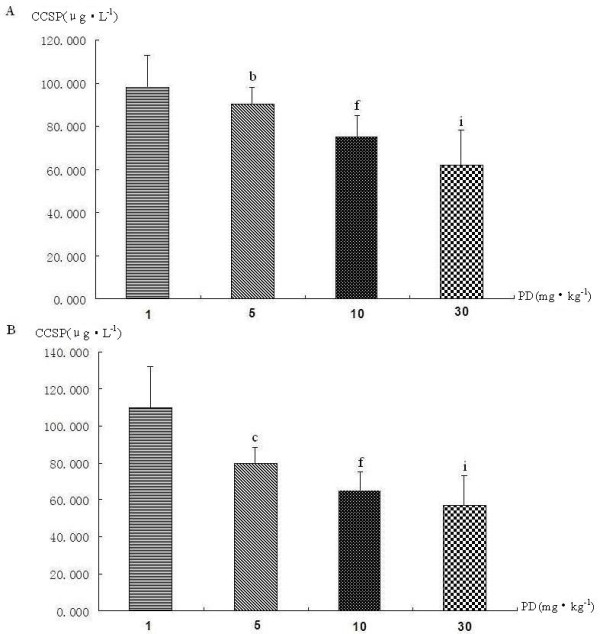
**Dose-effect relationship between polydatin and serum CCSP level in rat**. A.Serum CCSP level in PD treatment groups with different doses. B. Serum CCSP level in PD pretreatment groups with different doses. ^b^P < 0.05 and ^c^P < 0.01 vs. 1 mg·kg^-1 ^PD group, ^f^P < 0.01 vs. 5 mg·kg^-1^PD group, and ^i^P < 0.05 vs. 10 mg·kg^-1 ^PD group.

CCSP can transfer from lung tissue into the blood serum. Serum CCSP level is closely related to its synthesis and secretion by Clara cells in the respiratory tract, and serum CCSP level may act as a marker of the bronchoalveolar blood-gas barrier integrity and permeability [27,28]. As shown in Figure 9, serum CCSP level in the endotoxin shock group was significantly higher than in the sham operation group(Figure 9, group II vs. group I). However, CCSP levels in the PD treatment and PD pretreatment groups were lower than in the endotoxic shock group (Figure 9, group III/IV vs. group II). Serum CCSP level decreased with increasing PD dose (Figure 10A/10B). Therefore, PD decreased CCSP transference from lung to serum in a dose-dependent manner.

### Polydatin increases the percentage of CCSP-positive cells

In rat lung slices, CCSP-positive cells are non-ciliated epithelial cells, namely, Clara cells. The relative CCSP-positive airway epithelial cell proportions were as follows. Compared with the sham operation group, CCSP-positive cells were decreased in the endotoxic shock model group and increased in the PD control group (Table 4). Compared with the LPS group, CCSP-positive cells were increased in the PD treatment and pretreatment groups (Table 4). Thus, PD increased CCSP-positive cells in lung tissue in a dose-dependent manner (Table 5).

**Table 4:** Percentage of CCSP-positive cells of lung tissue in each group ( ± s, n = 10).

Group	The percentage of CCSP positive cells
I	20.3 ± 2.4
II	13.2 ± 1.3^b^
III	37 ± 4^be^
IV	46 ± 6^cf^
V	53 ± 6^cf^

I: sham operation group; II: endotoxic shock group; III: PD treatment group; IV: PD pretreatment group; V: PD control group. ^b^P < 0.05 and ^c^P < 0.01 vs. group I; ^e^P < 0.05 and ^f^P < 0.01 vs. group II.

**Table 5:** Percentage of CCSP-positive cells of lung tissue in each group ( ± s, n = 6).

Group	Dosage (mg·kg^-1^)	Percentage of CCSP-positive cells
PD pretreatment	1	40.2 ± 2.3
	5	54 ± 3^b^
	10	63.5 ± 2.6^f^
	15	79 ± 3^i^
PD treatment	1	37.4 ± 2.4
	5	46 ± 3^c^
	10	56.8 ± 2.4^f^
	15	70 ± 4^j^

^b^P < 0.05 and ^c^P < 0.01 vs. 1 mg·kg^-1 ^PD group, ^f^P < 0.01 vs. 5 mg·kg^-1^PD group, ^i^P < 0.05 and ^j^P < 0.01 vs. 10 mg·kg^-1 ^PD group.

### Polydatin up-regulates CCSP expression in BEAS-2B

**Figure 11:**
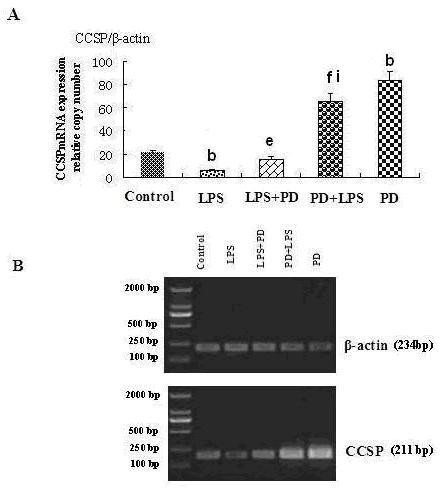
**Effects of polydatin on CCSP mRNA expression in LPS-induced BEAS-2B cells**. A. Relative expression of CCSP quantified by real-time PCR. B. RT-PCR analysis shown by electrophoresis. ^b^P < 0.05 vs. normal control group; ^e^P < 0.05 and ^f^P < 0.01 vs. LPS group; ^i^P < 0.05 vs. LPS+PD group.

**Figure 12:**
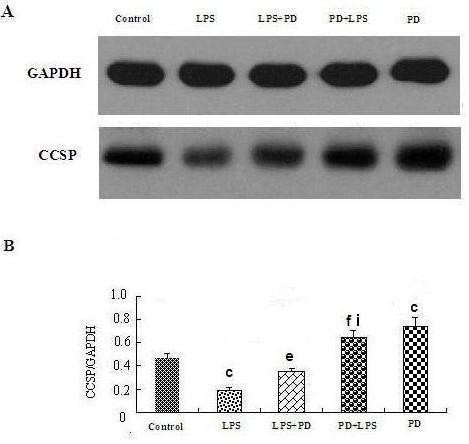
**Effects of polydatin on CCSP protein levels in LPS-induced BEAS-2B cells**. A. The electrophoretogram of CCSP protein expression. B. Relative ratio of CCSP protein expression. ^c^P < 0.01 vs. normal control group; ^e^P < 0.05 and ^f^P < 0.01 vs. LPS group; ^i^P < 0.05 vs. LPS+PD group.

The MTT assay suggested that the most suitable concentration of PD to use in BEAS-2B cells was 0.5 mmol/L and the most suitable concentration of LPS was 10 ng·ml^-1^. In vitro, LPS led to reduced CCSP expression in BEAS-2B cells, while PD promoted CCSP expression, not only in normal BEAS-2B cells but also in LPS-induced BEAS-2B cells (Figure 11 and 12). In addition, CCSP expression in the PD pretreatment group was greater than in the PD treatment group (Figure 11 and 12).

## Discussion

There are many experimental models of acute lung injury, but none of them completely reproduces all the prominent features of human ALI/ARDS, such as alveolar neutrophilia (indicating inflammation), hyaline membrane deposition (indicating disruption of the alveolar-capillary barrier) and microthrombi (indicating endothelial injury). Hence, there is no single "best" model of lung injury; instead, the best model will be one that reproduces the features to be tested in the investigator's hypothesis.

We used one of the well-established rat models of acute lung injury, intravenous administration of LPS because it can lead to activation of various inflammatory mediators such as phospholipase A_2_. These mediators may cause epithelial lesions and increase the alveolocapillary barrier permeability. Therefore, this model met all the requirements for our purpose.

First, we observed a reduction of CCSP mRNA in parallel with decreased CCSP protein expression in rat lung tissue after LPS stimulation for five hours. Prior to this, we knew that different inflammatory stimuli result in different CCSP expression patterns in lung. Inhalation of O_3 _and injection of phenyl isothiocyanate can induce acute and chronic inflammation in humans and increase CCSP expression in lung [27]. By contrast, CCSP production is decreased during acute and chronic lung injury caused by viral and bacterial infections, smoking [29] or chronic obstructive pulmonary disease. All of these factors make the role of CCSP in the inflammatory process more complex. Moreover, CCSP expression may result in tissue damage caused by inflammatory factors, rather than the inflammation itself [30]. It is most likely that up-regulation of CCSP expression is a result of the body's self-protection mechanism in the early stages of the inflammatory response, but suppression of CCSP expression follows during the later inflammatory response. CCSP expression is down-regulated after acute lung inflammation induced by intratracheal or intravenous LPS administration [28,31]. Our results are in agreement with these previous experimental observations. The finding that LPS reduces CCSP expression should provide an excellent foundation for future research.

Second, polydatin increased the CCSP-positive cell percentage and up-regulated CCSP mRNA and protein expression in lung tissue during acute lung injury induced by LPS. CCSP expression was increased along with PD increasing. There was a dose-effect relationship between PD and CCSP expression within our observational dose range. Additionally, CCSP protein expression was consistent with its mRNA level. Some possible mechanism by which PD promotes CCSP expression can be explained as follows: 1) PD can increase the number of CCSP-positive cells (i.e., Clara cells); 2) PD can promote the secretion of CCSP in individual Clara cells to increase total CCSP expression; and 3) most importantly, PD can enhance the stability of CCSP. The reduction of CCSP degradation is an important factor in increasing CCSP level. PD can help to keep the bronchoalveolar blood-gas barrier integrity and decrease the bronchoalveolar blood-gas barrier permeability induced by LPS so that CCSP will not be decomposed in serum. Berg et al [32] have reported that glucocorticoids regulate the CCSP promoter via C/EBP beta and -delta in lung cells. Ramsay et al. [33] have indicated that IFN-γ can up-regulate the expression of CCSP in rat and rabbit lung tissue. The reported mechanisms are as follows: 1) regulation of the Th1/Th2 cytokine network to increase CCSP mRNA expression; 2) hormone receptor and IFN-γbinding sites exist in the 5'-flanking region of the CCSP gene, which can directly promote the transcription of the CCSP gene and increase the synthesis and secretion of CCSP; and 3) increased stability of CCSP mRNA at the post-transcriptional level. Therefore, our future research will be focused on the effect of polydatin on the transcription of CCSP.

Third, CCSP expression in lung in the PD pretreatment group was greater than in the PD treatment group. This result suggests that a competitive inhibition of CCSP exists between PD and LPS. Therefore, we propose that PD and LPS have a common binding site in Clara cells that can influence the secretion of CCSP. However, the specific mechanism remains unclear, and the intrinsic link between PD and CCSP requires further investigation.

Fourth, based on animal experiments, the BEAS-2B cell line was chosen as the model to study the effect of PD on CCSP in vitro. BEAS-2B cells are clara cells that have been transformed by the SV40 T-antigen. BEAS-2B is the most commonly used model for CCSP research [34,35]. The human CCSP gene exists as a single copy in BEAS-2B cells. Tumor necrosis factor-α (TNF-α) can induce a change in chromosome structure that enhances the stability of an RNA-binding protein involved in transcriptional regulation, which increases CCSP gene expression in BEAS-2B cells [36]. Kim et al. [37] have reported that IL-13 and other indirectly acting proteins can promote the expression and activation of epidermal growth factor receptor (EGFR) so as to increase CCSP expression in airway epithelium. We found that CCSP can be expressed in and secreted by BEAS-2B cells in culture medium. LPS can decrease CCSP expression in BEAS-2B cells, while PD can promote the expression of CCSP in normal and LPS-stimulated cells, and the change in mRNA is consistent with CCSP protein expression. PD may enhance the proliferation of Clara cells and it may enhance the proliferation of CCSP-positive/pro-surfactant protein C pro-SPC-positive cells [38].

As far as the limitation of this research work was concerned, we just observed that polydatin suppressed PLA_2 _of lung induced by LPS through up-regulating Clara cell secretory protein. The intrinsic relationship between PD and CCSP need further investigation.

## Conclusion

We have demonstrated that PD can modulate CCSP expression to inhibit PLA_2_. This inhibition is probably a crucial protection mechanism of PD in LPS-induced acute lung injury. These findings provide compelling evidence for the potential efficacy of PD in clinical use, although the molecular mechanisms of its action need further exploration.

## Authors' contributions

SYS conceived the experiments and wrote the manuscript. ZYL determined the protein expression. MY determined the gene expression. LB did the animal model. LJW cultured the BEAS-2B cells. TBZ contributed to do the cell experiment. JC performed the immunohistochemical analyses and statistic analysis. TYL edited the draft and contributed significantly to experimental design. All authors have read and approved the final manuscript.
